# Physiotherapy Rehabilitation Following Acoustic Neuroma Resection in a Patient With Cerebellopontine Angle Tumour: A Case Report

**DOI:** 10.7759/cureus.54208

**Published:** 2024-02-14

**Authors:** Ishwin Kaur B Bagga, Subrat Samal

**Affiliations:** 1 Musculoskeletal Physiotherapy, Ravi Nair Physiotherapy College, Datta Meghe Institute of Higher Education and Research, Wardha, IND

**Keywords:** cawthorne-cooksey exercise, schwannomas, karnofsky performance status scale (kps), functional assessment of cancer therapy-brain (fact-br), functional assessment of cancer therapy-general (fact-g), vestibular ocular reflex exercise, physiotherapy rehabilitation, cerebellopontine angle tumor

## Abstract

Acoustic neuroma excision in patients with cerebellopontine angle (CPA) tumours offers particular rehabilitation problems due to the complicated architecture of the cerebellum and brainstem tissues involved. CPA tumours (acoustic neuromas) are slow-growing tumours that arise from the vestibulocochlear nerve. Surgical excision of these tumours can cause neurological abnormalities that compromise motor coordination, balance, and facial nerve function. The case study emphasises the importance of a comprehensive physiotherapeutic approach in rehabilitating a patient following acoustic neuroma excision, with a focus on particular CPA tumour deficits. The rehabilitation programme focuses on improving functional outcomes through balance, proprioception, and vestibular rehabilitation that is customised to the demands and deficiencies of the patient. Our comprehensive approach seeks to improve patients' quality of life, promote neurological healing, and support easy reintegration into normal activities following CPA tumour surgery.

## Introduction

The cerebellopontine angle (CPA) is a triangular region within the posterior cerebral fossa. Its superior borders include the tentorium, the brainstem, and the petrous section of the temporal bone [1,2]. It is occupied by the CPA cistern, which originates the cranial nerves V, VI, VII, and VIII, as well as the anterior inferior cerebellar artery [1,2]. The clinical importance of CPA arises from the wide range of lesions that affect this region and cause symptoms like hearing loss, tinnitus, and dizziness [1,3]. The two main varieties of CPA tumours are the bilateral acoustic neuromas linked to neurofibromatosis type 2 and the unilateral sporadic variation. Both tumour types are benign, develop slowly, and have a low risk for malignancy [1,4]. Schwannomas are frequently primary cranial nerve lesions, and meningiomas, epidermoid tumours, and arachnoid cysts are examples of arachnoid cysts that arise from misplacement, growth, or cyst formation of arachnoid cells [2,4,5].

Conditions that manifest as a variety of symptoms, including vertigo, headaches, hearing loss, tinnitus, dizziness, and aberrant gait, are usually associated with lesions in the CPA. Sensorineural hearing loss is caused predominantly by the damaged cochlear nerve, resulting in symptoms on one side [1]. Microsurgery, radiation therapy, or observation is the treatment choice for CPA tumours. Radiation therapy has various side effects like cranial neuropathies and worsening of hearing status [1,3]. Surgical procedure is very difficult in this case due to the complicated anatomy and narrow operative area [6]. Microsurgery is a treatment of choice as it preserves hearing and facilitates complete tumour removal. Surgical approaches available are translabyrinthine, middle fossa, and retrosigmoid approach, which is selected based on the size of the tumour [7-9].

Following surgical surgery for CPA tumours, physical therapy is a helpful therapeutic option [10]. The rehabilitation strategy mainly focuses on balance and coordination training as these are the factors that are majorly affected [11]. Vestibular ataxia and tinnitus require a proper rehabilitation and physiotherapy protocol [12]. Vestibulocochlear nerve damage requires balance training and coordination exercise training [13,14].

## Case presentation

Patient information

We outline the case of a 45-year-old female patient who was apparently alright six months back; then, she started complaining of dizziness, headache, urge to urinate often, and slightly blurred vision, for which she went to the local hospital and was managed conservatively. Due to the persistence of symptoms, she came to Acharya Vinoba Bhave Rural Hospital on December 8, 2023. She also has a history of decreased hearing in the right ear for the past eight years, along with feelings of general fatigue and weakness while doing work. The patient also has a history of kharra chewing for the past 17 years. She was admitted to the neurosurgery ward for the complaints, where she experienced a convulsion. She was then transferred to the neurointensive care unit. The patient required intubation via tracheostomy to maintain saturation. A planned physiotherapy protocol was made for the patient considering all the complaints and symptoms.

Clinical findings

The patient's informed consent was taken. The patient exhibited a mesomorphic physique and presented with tachypnoea and tachycardia, indicative of an elevated respiratory rate and heart rate. Positioned in supine with the head elevated at approximately 45°, the patient had an in-place Ryle tube, Foley catheter, and a central line. Evaluation using the Glasgow Coma Scale (GCS) recorded a score of E3VTM4, indicating a state of altered consciousness. Neurological examinations identified reduced biceps and ankle jerk on the right side, absence of plantar response, and diminished corneal reflex. The bilateral pupillary light reflex was observed to be sluggish, and the patient lacked a cough reflex. Cranial nerve assessments revealed impairments in the cranial nerves, trigeminal, abducens, facial, and vestibulocochlear, affecting mastication, lateral rectus muscle movements, facial expressions, and auditory acuity, respectively. The patient was mechanically ventilated via tracheostomy in continuous mandatory ventilation mode due to aspiration, with a fraction of inspired oxygen (FiO_2_) at 50%, positive end-expiratory pressure (PEEP) at 7 cmH_2_0, and supplemental oxygen via T-piece at 6 litre. Functional ability assessment revealed the patient's ability to roll on the bed with maximum assistance.

Diagnostic assessment

**Figure 1:**
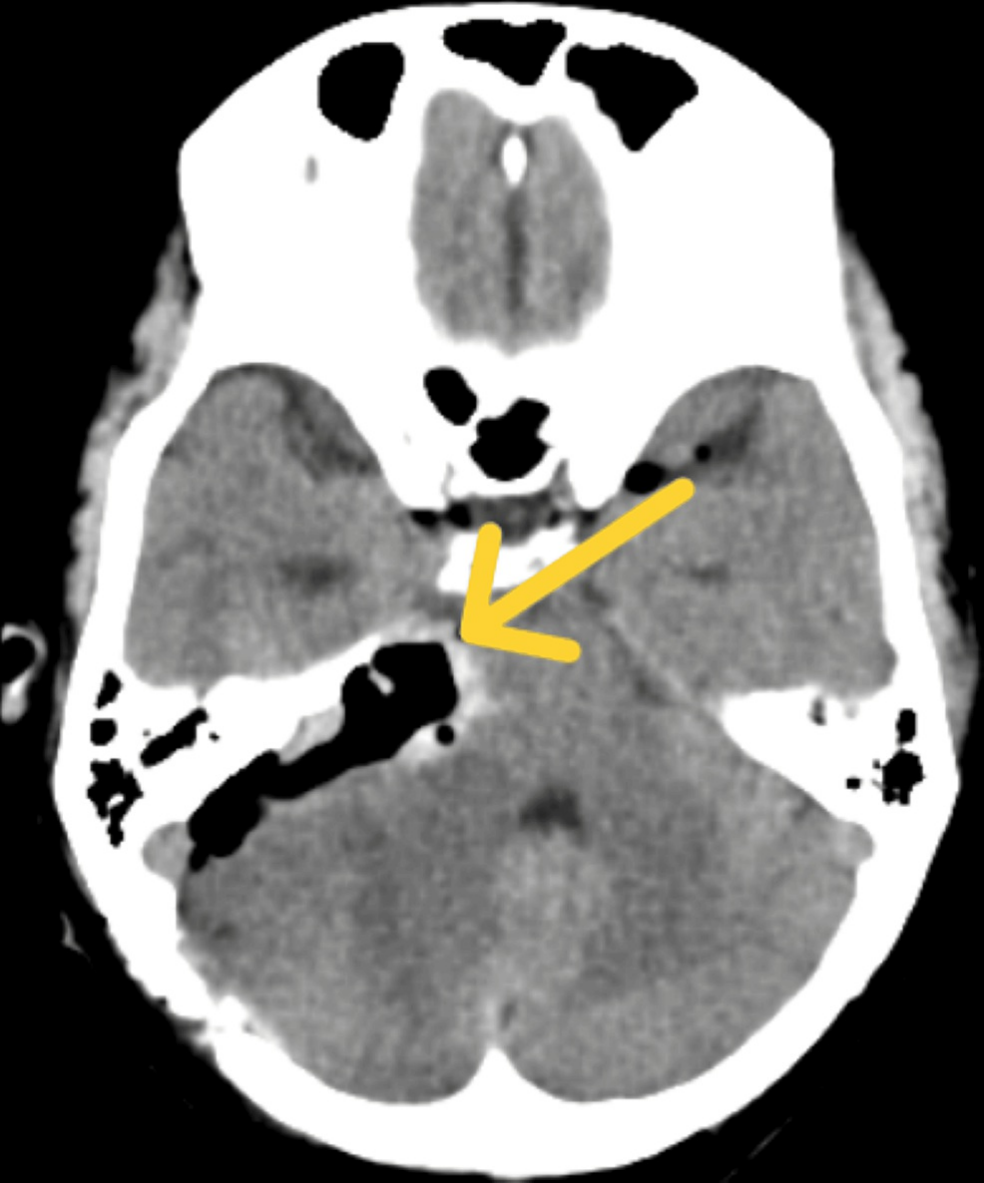
CT scan of the patient showing tumour resection The yellow arrow shows the site of tumour resection. CT: computed tomography.

Diagnostic investigations like complete blood count (CBC), liver function test (LFT), and C-reactive protein (CRP) were done, which were returned to normal ranges. A kidney function test (KFT) indicated hyponatremia. A computed tomography (CT) scan showed a post-operative calvarial defect in the left occipital bone with overlying extra-calvarial soft tissue swelling. The arterial blood gas (ABG) analysis in the patient revealed an uncompensated respiratory alkalosis. Figure 1 shows the postoperative CT scan.

Physiotherapy intervention

Table 1 describes the planned physical therapy rehabilitation protocol which was made for the patient.

**Table 1:** Physiotherapy rehabilitation protocol planned for the patient

Problem list	Cause	Goals	Intervention	Rationale
Dizziness	Compression on cerebellum or brainstem	To reduce the dizziness in patient	Cawthorne-Cooksey exercises: to minimize the symptoms of dizziness. Turn head forward and backward and side to side. Repeat four to five times per day.	Prevent dizziness and improve general health.
Blurry vision	Pressure on optic nerve	To reduce the symptoms associated with patients’ condition	Vestibular ocular reflex exercise: to minimize the symptoms of dizziness and blurry vision. Should be repeated five times per day.	Help patient to focus correctly and help improve vision.
Vertigo	Lesion of vestibular nerve	To reduce the episodes of vertigo and intensity	Cawthorne-Cooksey exercises to be given to reduce the symptoms of vertigo for 8-10 mins on regular basis. This involves moving head upwards, downwards, and sideways with some force.	Physiotherapy aims not only to reduce the symptoms of vertigo but also to enhance overall functional ability.
Fatigue	Reduced sleep, anxiety, stress	To maintain the fatigue of patient	Educate the patient. Teach patient to prioritize activities and break them into smaller, more manageable tasks.	Target specific muscle groups affected by weakness due to the tumour. Enhance overall functional strength to improve daily activities.
Breathing difficulty	Prolonged immobility	To prevent breathing complications	To prevent chest complications, deep breathing exercises, dyspnoea reliving positions, etc.	Help to prevent chest complications and improve breathing and lung capacities.
Muscle weakness	Tumour causes changes in neural pathways making generalised muscle weakness	To increase the overall muscle strength	Isometric exercises help maintain muscle strength without causing excessive strain on the neck. Strengthening exercises to upper limbs and lower limbs to improve mobility. Use of Therabands and weight cuffs, etc. 10 repetitions each.	Target specific muscle groups affected by weakness due to the tumour. Enhance overall functional strength to improve daily activities.
Decreased range of motion	Potential post-surgical limitations	To improve and restore functional ranges of joints	Stretching to the muscles to loosen muscle tightness. Core stability exercises help improve posture, reducing strain on the neck and supporting structures. Five sets of 20 repetitions.	Prevent contractures and improve joint flexibility, especially in post-surgical cases.
Decreased functional mobility	Impact on daily activities due to tumour and treatment effects	To enhance the functional mobility of patient	Progress to more functional activities as the patient's condition allows focusing on independence.	Focus on activities relevant to the individual's daily life to improve overall independence.
Balance and coordination impairment	Ataxia, vestibular dysfunction	To improve balance and coordination	Exercises like mini wall squats, tandem standing and walking, training on parallel bars, Swiss ball balancing, spot marching, and walking sideways in both directions. To be done four to five times daily.	Improve proprioception and coordination, addressing ataxia and vestibular dysfunction.
Gait disturbances	Lesion at the junction of the cerebellum and the pons	To improve the gait and gait pattern	Gait training (twice a day) is given in which postural correction exercises, balance training, and gait training exercises are initiated.	Help to improve the walking patterns and address any gait abnormalities caused by the tumour or medical treatment.

Outcome measures

**Figure 2:**
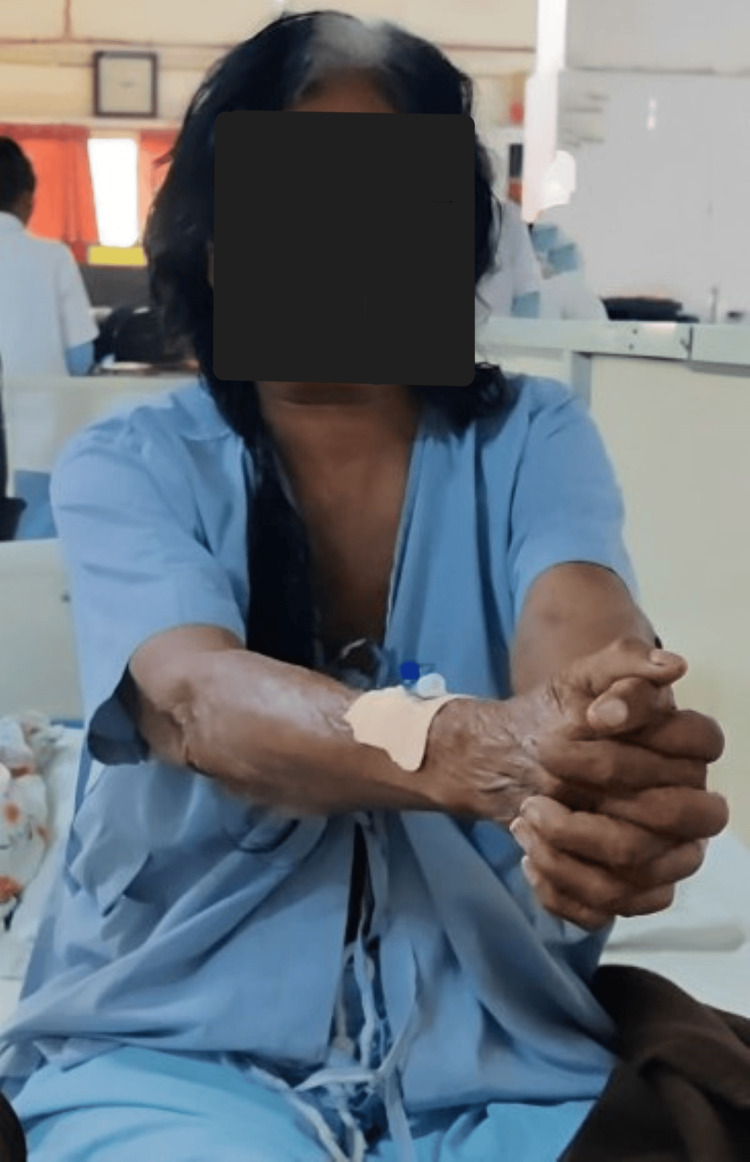
Thoracic expansion exercise given to the patient

**Figure 3:**
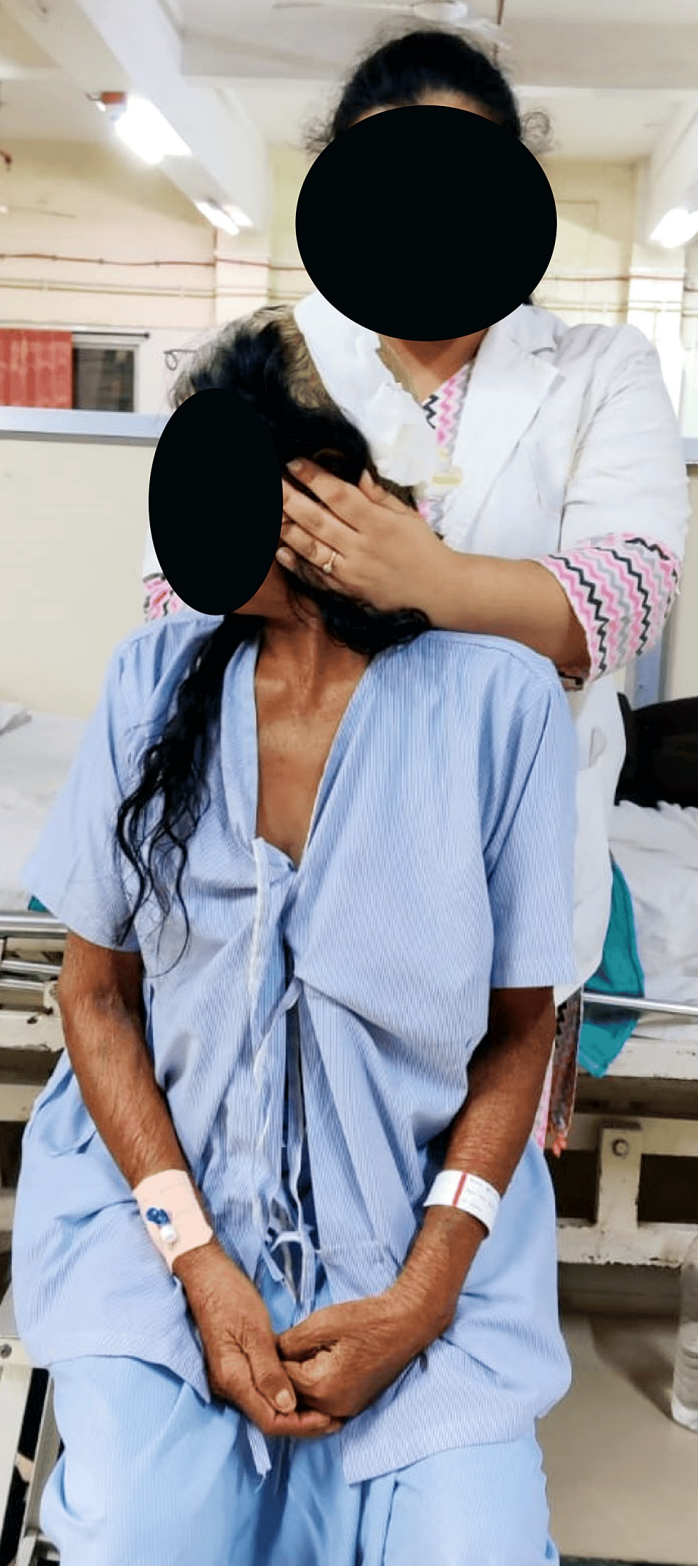
Head movement exercises given to reduce vertigo symptoms

**Figure 4:**
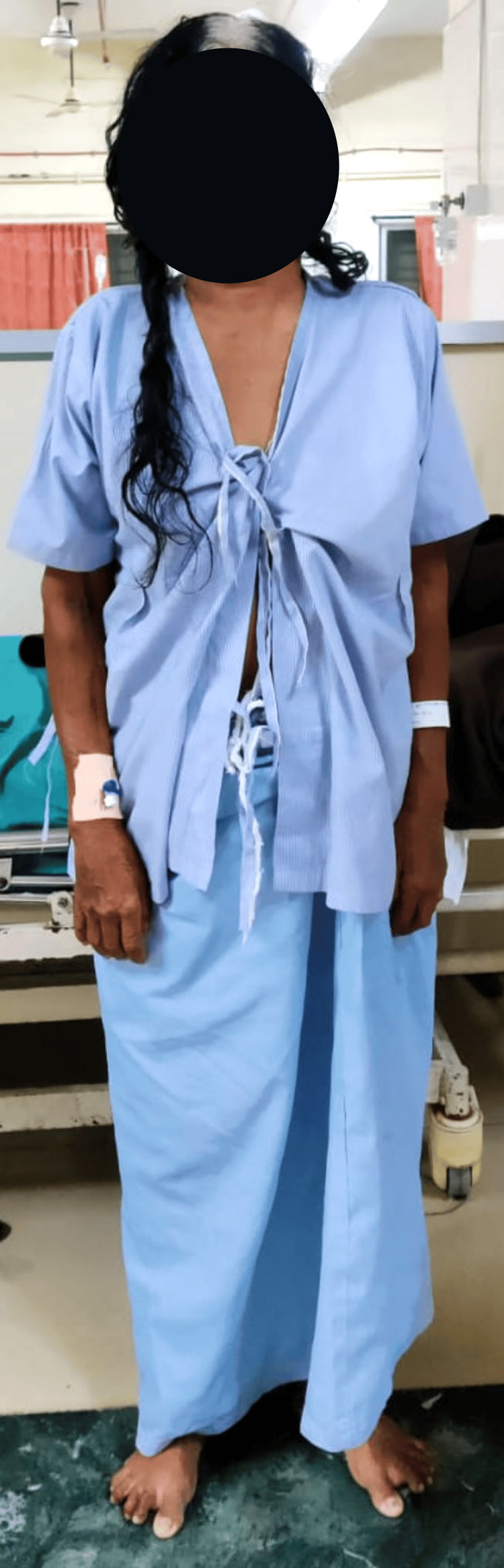
Sit-to-stand initiated for the patient

Table 2 shows the outcome measures that were used to assess the patient’s progression post-rehabilitation protocol. Figure 2 shows the thoracic expansion given to the patient. Figure 3 shows head movement exercises given to reduce vertigo symptoms. Figure 4 shows sit-to-stand.

**Table 2:** Outcome measures KPS: Karnofsky Performance Status Scale, FACT-BR: Functional Assessment of Cancer Therapy-Brain, FACT-G: Functional Assessment of Cancer Therapy-General, FIM: Functional Independence Measure.

Component	Pre-rehabilitation	Post-rehabilitation
KPS	Score=30% (severely disabled, unable to care for self and requires hospital admission and care)	Score=70% (unable to do active work and requires assistance in some activities, cares for self, and some symptoms are seen)
FACT-BR	50/92 (patient had maximum dependency)	42/92 (patient has minimum dependency only in a few tasks)
FACT-G	Physical well-being: 28/28; social/family well-being: 19/28; emotional well-being: 20/24; functional well-being: 4/28	Physical well-being: 12/28; social/family well-being: 21/28; emotional well-being: 7/24; functional well-being: 14/28
FIM	Score=68/126 (severe dependency)	Score=112/126 (mild dependency)

## Discussion

The CPA tumours are common in adults and require physiotherapy treatment postoperatively [15]. CPA tumours account for 5%-10% of all intracranial neoplasms [2,16,17]. Physiotherapy plays a vital role in balance and coordination training, gait training, endurance training, and in reducing the symptoms related to CPA tumours [13,18]. Physical therapy rehabilitation plays a major role in the restoration of balance and improving balance, hence reducing the risks of falls [19,20]. In this case, the patient complained of inability to balance, weakness of the muscles of the face, and early fatigue. Physiotherapy rehabilitation focuses on gait training, balance training, facilitating the sit-to-stand for the patient, improving the strength of the patient, etc. These encouraged the faster recovery of the patient and helped to prevent secondary complications in the patient. Various studies show the importance of physiotherapy postoperative in cases of CPA tumours. Exercises focusing on vestibular rehab help to improve postural stability significantly and reduce vertigo. In this case study, it is found that physical therapy rehabilitation enhances recovery and improves the overall quality of life of the patient.

## Conclusions

It is concluded that physiotherapy rehabilitation post-excision of CPA tumours is very beneficial to patients. The rehab focuses on all the symptoms and postoperative complications and gives a corrective way to them. The patients’ strength and mobility improved resulting in an improvement in functional ability. The symptoms like blurry vision, vertigo, and dizziness were also reduced with the help of the planned protocol for the patient. The patient understood the importance of physiotherapy and the long-term effects of the exercises. It helped the patient to improve her overall health and her quality of life improved significantly.
